# Supplementary material for: Learning curve and surgical outcome of robotic assisted colorectal surgery with ERAS program
Source: Sci Rep. 2022 Nov 29;12:20566. doi: 10.1038/s41598-022-24665-w (PMC9709162; doi:10.1038/s41598-022-24665-w)
Supplement: Supplementary file 2 — Supplementary Information 2. [file 41598_2022_24665_MOESM2_ESM.zip › RAL Raw data-Ñ[▒K/107/OP_ñw╝╨░O▒Kñσ.pdf]

正式摘要(電子簽章：000000223603743)

W52 / W52

臺中榮民總醫院

Operation Note

[redacted]

- 手術日期：2021/08/20
- 手術醫師：VS 林俊余，CR 陳映菁，R 郭連雄，R 陳瑋夫
- Anesthesia : GE
- Position : Lithotomy
- Aseptic agents : Soap , Betadine Aqua

Diagnosis

- Pre-operative diagnosis : upper rectum cancer 10 cm FAV.
- Post-operative diagnosis : upper rectum cancer 10 cm FAV.
  s/p robotic assisted low anterior resection
  cT3N1M0

Surgical Indication

- For removing the primary tumor

Operation

- 1. Robotic (XI) assisted anterior resection
  2. Intraoperative colonoscopy : (-).
  3. High ligation (+) : At IMA root. Left colic artery and IMV were
  also ligated
  4. Indocyanine green agent was used to check the blood supply of the colon
  25mg/10cc, proxiaml: 3cc, 58 sec, distal: 3cc, 52 sec
  5. Take down from the splenic flexure : (-)
  6. Robotic endoGIA (45-4.3) X2 + EEA (29mm) were used
  7. Proximal cut end: 8 cm, distal cut end: 2 cm
  8. Hemovac drainage tube in pelvis.

Finding

- 1. A 4 x 4 cm tumor at S-colon
  2. No other gross tumor nor polyps was found. There was no obvious visible liver
  metastasis, no visible peritoneal seeding. No obvious ascites was noted
  3. High ligation (+) : At IMA root. Left colic artery and IMV were
  also ligated
  4. Indocyanine green agent was used to check the blood supply of the colon
  25mg/10cc, proxiaml: 3cc, 58 sec, distal: 3cc, 52 sec
  5. Take down from the splenic flexure : (-)
  6. Robotic endoGIA (45-4.3) X2 + EEA (29mm) were used
  7. Proximal cut end: 12 cm, distal cut end: 2 cm
  8. Hemovac drainage tube in pelvis. Hyalobarrier+
  9. Bilateral doughnuts : intact ; Air leak test: Done and no air leak
  was found

Procedure

- 1. General endotracheal anesthesia was induced and then the patient was placed in modified
  Trendelenburg lithotomy position.
  2. Preoperative antibiotics were given. A Foley catheter and nasogastric tube were placed.
  3. The abdomen was prepped and draped in standard sterile fashion.
  4. A transverse incision was made over the right side of the umbilicus and a 12 mm trocar was

- inserted into the peritoneal cavity by open method. Peumoperitoneum was established with CO2 inflation.
  5. Laparoscopy was inserted and then other two 8mm trocars were inserted over LUQ area of abdomen, also another one over RLQ area of abdomen. Finally, a 12mm trocar was inserted over RUQ area as an assist-port. The operative findings were mentioned as above. After the setting was completed, the laparoscopy was shifted to the LUQ 8mm trocar site instead.
  6. The sigmoid colon was held by grasper and the peritoneum overlying the mesentery was scored with electrocautery.
  7. The inferior mesentery artery was identified, ligated above the left colic artery with endoclips, and transected.
  8. Dissection was done above the Gerota's fascia and toward the peritoneal attachments.
  9. The colon was mobilized from retroperitoneum overlying the sacral promontory and into the pelvis.

  10. Peritoneal reflection was not resected.
  11. Distal descending colon and s-colon were freely mobilized after the mesoclon was all dissected.
  12. Indocyanine green agent was administered from IV line. The blood supply of the colon was examed carefully.
  12. The distal s-colon was divided with Robotic endoGIA (45-4.3) X2.
  13. A new incision was made over LLQ area of abdomen. We approached into the peritoneal cavity by open method. S-colon was pulled out.
  14. The proximal colon was then taken out for carefully inspection, and Indocyanine green agent was administered again from IV line, to make sure it has an excellent blood supply. The mesenteric fat was gently cleared from the terminal 2-3 mm of the bowel ends.
  15. The field was isolated with moist pad and the bowel opened and sized. It was determined that a 29 mm circular stapler could be accommodated. A purse string suture of 2-0 prolene was placed. The tumor-containing tissue was removed. Then the anvil was inserted, followed by the purse string suture. The colon was then put back to the abdominal cavity.
  16. Pneumoperitoneum was constructed again.
  17. The anal canal was irrigated with distil water and then the circular stapler was introduced through the anus and pierced through the upper area (The ventral site of the body) of the stapler line.
  18. The proximal bowel with anvil in place was engaged with the circular stapler and the stapler was then fired. Two complete donuts were obtained and the distal one was submitted to pathology for margin confirmation.
  19. The anastomosis was tested for leaks with irrigation solution and air introduced through the rectum and was found to be patent and intact.
  20. The fascia of 12mm trochar sites and LLQ extended wound were closed with interrupted sutures via Num.1 Vicryl.
  21. The wound was further closed with 3-O Vicryl, and the skin was applied with dermabond.
  22. The patient tolerated the procedure well and was brought to the POR in stable condition.
- Estimated blood loss: minimum
- 器械數量: 正確
- 紗布數量: 正確

Image

- PAINT
  - colon & rectum

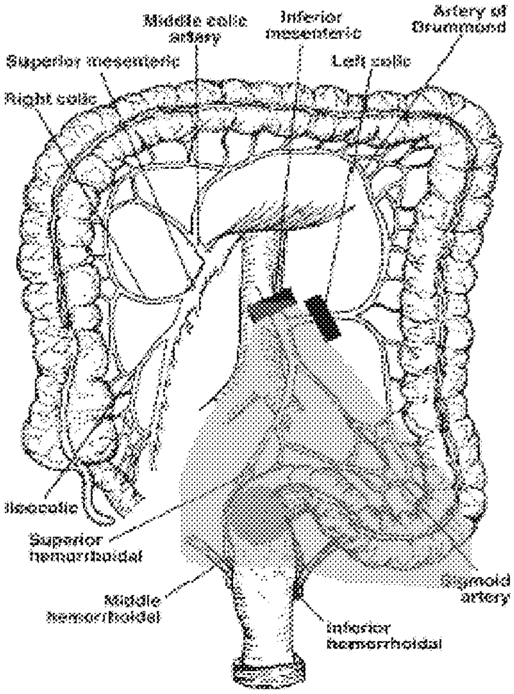

編輯主治 2222H林俊余

正式摘要**(電子簽章：000000223603743)**

W52 / W52

doctorRank:B
